# Wisdom from the past

**Published:** 1970-04

**Authors:** 


### Wisdom from the Past

### Extracts From This Journal — 1895 and 1920

### SEVENTY-FIVE YEARS AGO

**From a Review of "The Vaccination Question". By Arthur Wollaston Hutton. Pp. 128. London: Methuen & Co. 1894.**

"We have read a great many strange things, and we have heard a great many wild statements; but we do not ever remember before coming across an intelligent man like Mr. Hutton, more assertive of doing strict justice to a subject, and at the same time displaying more prejudice and preconceived ideas. Vaccination is no good—and worse—because the author has determined that it is of no avail! he "will not be learned nor understand, but walks on still in darkness" (p. 70). We do not intend to deal with every point raised in this little work; if we can knock down, as we believe we can, some of his strongholds, the smaller outworks cannot well be held.

Jenner, like most earnest and enthusiastic men, did unquestionably maintain the life efficacy of vaccination. Time has gone on and has shown that vaccination is not more potent against an attack of small-pox than small-pox itself. An attack of severe unmitigated small-pox does not invariably render a person immune to a second attack, or even a second attack to a third; but these cases are very rare, and the supporters of vaccination may admit that small-pox is, perhaps, rather more protective than vaccination and revaccination about the twelfth year: but the process is a very dreaded and dreadful one.

Then there is not a lot of proof that vaccination has any connection with syphilis, as some modern pseudo-scientists assert. McVail has shown conclusively that deaths from infantile syphilis are as frequent in Scotland during the first six months of life as in England; and yet vaccination is not carried out in Scotland until the sixth month and later. Whereas if vaccination were modified syphilis, the roll of deaths in England under six months, ought to outnumber those in Scotland but they do not; nay more, the averages for both countries continue as nearly as possible alike, through all ages. Then again, we never knew that people who had syphilis, or who had had it, derived any special protection against small-pox in the way that vaccination unquestionably does confer it, which surely ought to be the case were vaccine modified specific germs."

"In Table I let us take Bristol, with its (assumed) 5.2 per cent of vaccination default only, and compare it with Dewsbury with its 37.1 per cent default. The deaths in Dewsbury are given as 126 (but there are one-and-a-half years short), and in Bristol at 123 (over-stated): 123 in well-vaccinated Bristol. 126 in badly-protected Dewsbury! *Prima facie*, the latter very little worse off than Bristol! But, what an unfair, dishonest position! Dewsbury has, in round numbers, only a population of 30,000, whilst that of Bristol is 230,000. In a Dewsbury the size of Bristol, the deaths would have been not 126, but at the very least 126 × 7, or 882! In a Bristol the size of Dewsbury, the deaths would have been 123 ÷ 7, or 17 only.

Take Sheffield: Mr. Hutton states that deaths at 711, in a population of, say, 324,000, or nearly eleven times the size of Dewsbury. In the same ratio as Dewsbury, they would have been 1386. Or, again, to take the boasted Leicester. We will admit that the place has escaped, so far, a severe incidence of small-pox; but what is the mortality of vaccinated persons attacked, as compared with the unvaccinated? 1.1 per cent in the former, 15.8 per cent in the latter, according to most trustworthy figures published in the *British Medical Journal*. We may safely predict that Leicester will have its day of reckoning yet: and such a town not only shows a very bad example, but disperses a large number of unvaccinated persons far and wide, and thus inflicts most unfair conditions on other communities. After these statistics and their fallacies, we can appraise at its right value the "reasonable hypothesis of constitutional immunity" (p. 72), as an explanation of the freedom of revaccinated cases from small-pox, and laugh at the charge that vaccination is kept up by doctors in Harley Street (note p. 111), and the corporation of doctors generally for pecuniary interest. The gravamen is absurd in the extreme. It would be far more profitable for medical men to vaccinate and re-vaccinate themselves, their families, and friends, and then let small-pox run rampant.

Mr. Hutton is greatly to be blamed for the introduction of the almost blasphemous picture of calf-worship (p. 115), and, being librarian of a large political club, he ought to be specially censured for publishing a book without an index."

(Vol. 13, pp. 221-223, 1895)

**From a paper on "School Surgery" by Arthur W. Prichard.**

"In preparing the cricket part of my paper, although I do not wish it to be understood that he is in any way responsible for the opinion I have just expressed, I have asked to give me his experiences of accidents in the cricket field the greatest of living cricketers, who, among the many records that he has broken, holds the world's record—at all events, among medical men—of having his name known to the largest number of English-speaking people in the universe. He with us all rejoices at the immunity from serious injuries which cricket enjoys. One injury, he says, that occasionally occurs, and one which he has suffered from himself, is, that in starting from the crease to make a sharp run a few fibres give way in the gastrocnemius, making the player feel as if he had been hard hit on the back of the leg. This quite incapacitates the player for the time from running, and the best treatment, although very painful, is to have the part strapped with firm plaster, or to have an elastic bandage put on. Indeed, for many cricket injuries an elastic bandage is a very useful item in one's cricket bag. Mr. W. G. Grace has seen one death in the field, and that was a sad case of a boy at Harrow who received a blow on the head, while umpiring, from a ball hit to leg by a batsman of his own game, while he was paying too much attention to a more important game played close by. Another death, but not on the cricket field, occurred a few years ago in the Notts v. M.C.C. match. A professional player was stunned, but insisted next day in travelling home, saying he was not seriously hurt. Coma came on and he died before he reached home, presumably from haemorrhage. On August 22nd this year, a player at Clapham when batting was struck in the neighbourhood of the heart and died in a few minutes.

A curious case that Mr. Grace mentioned was that of a Kent gentleman, who, in playing at Gloucester against Gloucestershire, jumped at a catch, while fielding at point, and dislocated his shoulder. The following year, in the same match but at the County Ground, Bristol, he fell while fielding and met with the same result. Mr. E. M. Grace brought him at once to the Infirmary, where I happened to be on duty, and I reduced the dislocation. Notwithstanding that he had to be put fully under chloroform to effect the reduction, the Kent player insisted upon going back to see the match an hour after he had come round. One case Mr. W. G. Grace cited as showing the pluck of some players was this. A wicket-keeper dislocated the top joint of his finger so badly that the skin was split across. This was reduced and a bandage put on. He then resumed his place at the wicket, and did his work with credit till the end of the match. Bad blood-blisters are common, and sometimes he has seen broken metacarpal bones, but never any serious damage to the abdomen or to the scrotum. This last fact rather surprised me, as a blow upon this part of the body of the batsman or wicket-keeper is apparently so frequent."

(Vol. 13, pp. 252-253, 1895)

### FIFTY YEARS AGO

### Progress of the Medical Sciences.

"**Diverticulitis** is assuming a clinical importance in much the same manner as appendicitis did about thirty years ago. Though known to the few, it has not yet found its way to most of the ordinary text-books, and it has until recently been regarded by the surgeon only from two aspects—as the chief cause of vesico-colic fistula, and as a cause of stenosis of the large bowel resembling malignant disease.

The name is not all together satisfactory, as diverticula are congenital and acquired, and it is the congenital form with which most people are more familiar, but the acquired form which is affected in the condition under consideration. Dr. W. H. Maxwell Telling, who opened the discussion on this subject at a recent meeting of the Royal Society of Medicine, and whose remarks are taken as the basis of this review, practically restricts the term "diverticulitis" to the "inflammatory changes and secondary pathologic processes generally occurring in or in connection with a certain type of diverticulum. This type is the secondary, acquired, multiple false diverticula of the large bowel, particularly and nearly always found in the sigmoid flexure." It should, however, be borne in mind that acquired diverticula may occur in any part of the alimentary tract. Acquired diverticula are most common after sixty years of age, and it is probable that constipation is an important factor in their production. As Dr. Telling points out, the diverticula are often overlooked because of their smallness, because they mainly enter the fat-laden appendices epiploicae, and are not discoverable without careful dissection."

**Diagnosis.**—The condition which presents the greatest difficulty is the differentiation between carcinoma of the bowel and the peridiverticulitis which exists as a contracting tumour tending towards chronic intestinal obstruction. Even at the time of operation the diagnosis between these conditions may be impossible, but the following points Telling regards as being in favour of peridiverticulitis: (1) The absence of the "shadows of malignancy" from the general condition; (2) tendency to obesity, and maintenance of good nutrition generally; (3) long history of attacks of abdominal pain in the left lower quadrant; (4) history of tumour formation, with subsequent disappearance; (5) absence of blood (visible to naked eye) in stools over a prolonged period; (6) presence of vesical fistula, in which malignancy can be excluded by cystoscopy; (7) negative sigmoidoscopy as regards malignant disease; (8) X-ray demonstration of diverticula; (9) pyrexial attacks; (10) examination of blood showing the presence of neutrophilic leucocytosis, and the absence of the specific nuclear changes characteristic of cancer. Sigmoiditis, hyperplastic tuberculosis, actinomycosis, syphilis and pelvic conditions generally may require consideration in forming a diagnosis.

**Treatment** is by operation unless there is some special contraindication. All diverticulum-bearing gut should be removed, or recurrence may take place. There seems to be a special liability to post-operative peritonitis in some cases. Care should be taken in handling the gut, less rupture of a diverticulum should occur. No case of supposed carcinoma of the lower bowel should in future be regarded as inoperable unless diverticulitis has been fully considered."

James Swain.

(Vol. 37, pp. 57-59, 1920)

